# P63. Autologous tumour cells and SW742 allogeneic cell line have comparable stimulating effect on PBMCs of gastrointestinal malignant patients in vitro

**DOI:** 10.1186/2051-1426-2-S2-P37

**Published:** 2014-03-12

**Authors:** A Sheikhi, K Saadati, R Salmani, N Mousavinasab

**Affiliations:** 1 Dezful University of Medical Sciences, Immunology, Dezful, Iran; 2 Zanjan University of Medical Sciences, Surgery, Zanjan, Iran; 3 Zanjan University of Medical Sciences, Social Medicine, Zanjan, Iran; 4 Zanjan University of Medical Sciences, Immunology, Zanjan, Iran

## Background

Natural killer activity is believed to be important contributor of a patient’s immune system to fight cancer. However, cancer patients have reportedly defective NK activity and the malignant target frequently has developed mechanisms to escape detection of NK cells. Our research is aimed at overcoming this NK cell deficiency.

## Materials and methods

Malignant autologous epithelial cells of 10 colorectal carcinoma patients were separated by cell culture procedures. Peripheral blood mononuclear cells (PBMCs) were stimulated with their mitomycin treated autologous tumour cells or allogeneic SW742 colorectal carcinoma cell line. The expression of CD3, CD56, NKG2D and NKp44 were detected with flowcytometry and reverse transcription-PCR. NK activity of PBMCs against K562 target cell line was measured by MTT colorimetric assay.

## Results

Stimulation with autologous tumour cells and allogeneic SW742 colorectal carcinoma cell line augmented CD56+ and CD56+CD3+ cells and up-regulated NKG2D and NKp44 expression. NK activity of PBMCs after co-incubation with autologous tumour cells or SW742 was significantly raised.

## Conclusions

Our results demonstrated that stimulation of PBMCs by SW742 can significantly improve NK activity as much as by autologous tumour cells which was confirmed by the higher expression of NKp44 and NKG2D. Since the separation of autologous tumor cells is difficult and time consuming the allogeneic tumour cell line could be a good replacement for large scale short term generation of activated NK cells. These data may help to improve cancer immunotherapy protocols.

